# Simultaneous evaluation of iris area and subfoveal choroidal thickness in Fuchs uveitis syndrome

**DOI:** 10.1186/s12886-024-03304-0

**Published:** 2024-01-19

**Authors:** Matilde Ruiz-Cruz, Patricia Navarro-López, Gerardo Marcelo Hernández-Valero, Luz Elena Concha-del-Rio

**Affiliations:** 1 Inflammatory Eye Disease Clinic. Asociación para Evitar la Ceguera en México, Vicente Garcia Torres No. 46 Coyoacán, Mexico City, 04030 Mexico; 2 Asociación para Evitar la Ceguera en México, Vicente Garcia Torres No. 46, Coyoacan, Mexico City, 04030 Mexico

**Keywords:** Fuchs uveitis syndrome, Optical coherence tomography, Iris area, Choroidal thickness, Inter-eye differences

## Abstract

**Background/aims:**

To simultaneously evaluate iris area (IA) and subfoveal choroidal thickness (SFCT) in eyes with Fuchs Uveitis Syndrome (FUS).

**Methods:**

We prospectively recruited a case series of patients with FUS at our institution, simultaneously measuring IA with anterior segment spectral domain optical coherence tomography (SD-OCT) and SFCT with enhanced depth imaging optical coherence tomography (EDI-OCT). Iris images were analyzed by ImageJ software. We tested the differences in intereye IA and SFCT with the healthy eye (HE) using the Wilcoxon test, and clinical interpretation was controlled by intraclass correlation coefficient (ICC) between two masked specialists.

**Results:**

Sixteen patients with unilateral FUS were included. Six were female, and the age range was 37 to 67 (median age 48 years, IQR 41–60). ICC of 98.9%, with a lower confidence interval of 97%. Eyes with FUS had a significant thinning of the total iris median area (*p* < 0.002), restricted to the temporal and nasal areas compared to the HE (*p* < 0.01 and < 0.001, respectively). SFCT was also significantly thinner compared to the HE (*p* < 0.0001). A low correlation was found between iris and choroidal thinning in FUS eyes (r_s_ = 0.21; *p* = 0.4).

**Conclusions:**

This study found reduced iris area and subfoveal choroidal thickness in eyes with FUS compared to the normal fellow eye.

## Introduction

Fuchs Uveitis Syndrome (FUS) is an anterior, non-granulomatous, mild, chronic, and mostly unilateral uveitis [1, 2]. Diagnosis is based on clinical findings: iris heterochromia (hypochromia of the affected eye) due to iris atrophy, diffuse fine stellate keratic precipitates with fibrillary extensions, low-grade iridocyclitis, absence of synechiae, and presence or absence of vitreous inflammation [3]. 

The irises of patients with FUS show some loss of pigment epithelium and a decrease in anterior stromal thickness and volume, leading to heterochromia; the anterior stromal atrophy leads to the pigmented layer becoming dominant [3, 4]. Basarir et al. [5], using Anterior Segment Optical Coherence Tomography (AS-OCT), reported reduced iris thickness in eyes with FUS compared to healthy eyes (HE).

Ozer et al. [6] found a statistically significant difference in the optical density of the temporal iris pigment epithelium between the FUS eye and the HE. Invernizzi et al. [7], determined that Spectral Domain Optical Coherence Tomography (SD-OCT) provided a better image than time-domain AS-OCT for evaluating irises both in pathologic conditions and healthy subjects. They discovered that the temporal iris was thinner in eyes with FUS compared to HE.

Most studies have associated subfoveal choroidal thinning in eyes with FUS in contrast with the HE [8, 9], but one study has found opposite results [10]. There is still great discussion about the causes of reduced Subfoveal Choroidal Thickness (SFCT), FUS associated iris atrophy, and the etiology of FUS. A population-based study found that SFCT reduction was primarily associated with > 50 years of age, and other systemic physiologic factors did not seem to contribute significantly [11]. This contradicts the average age of onset of FUS [12], suggesting a different cause. The Rubella virus has also been suggested as a cause of FUS [13]. The overall inflammatory cytokine environment seen in most uveitis cases would concur with a viral etiology of FUS with SFCT and its reductions. If there is a common underlying trigger for FUS, it would be expected that both measurements made here would be equally affected [14]. 

From a methodological standpoint, pathological effects on iris thickness and SFCT are complicated to study, since various genetic and geographical factors influence what is considered “baseline” thickness for each population [12, 15, 16]. Differences can be found even within populations with Amerindian and European ethnic background, so called “Mestizo” ethnicities [15, 17]. Explanations related to histocompatibility and virus affinity have been proposed [18, 19], but, currently, there is no consensus for this high inter- and intrapopulation variability. The purpose of our study was to compare iris thickness (area) with SFCT in patients with unilateral FUS, allowing each patient to be their own control.

## Materials and methods

This study was conducted at the Asociación para Evitar la Ceguera en México IAP, Hospital Luis Sánchez Bulnes, in Mexico City, between June and November 2019. All patients with a diagnosis of unilateral FUS based on clinical examination of the Kimura criteria and classification criteria for FUS [3, 16, 20] (keratic precipitates, anterior chamber cells, iris atrophy with or without heterochromia, and absence of synechiae) were invited to participate. Exclusion criteria were cases in which the posterior pole could not be reviewed because of lens or corneal opacities, bilaterally affected cases or cases with previous non-surgical trauma. The study was approved by the Institutional Review Board and Ethics Committee of Asociación para Evitar la Ceguera en México, I.A.P, following Mexican law, and all methods were carried out in accordance with Declaration of Helsinki, study protocol number UV-21-04. Written informed consent was explained and signed by all participants.

Patients underwent a complete ophthalmic examination in both eyes, including Best corrected visual acuity (BCVA) determined using the Snellen test, and the obtained values were converted to logarithm of the minimum angle of resolution (LogMAR) for statistical purposes; bio-microscopic anterior segment (iris characteristics and anterior chamber inflammation grading measured according to Standardization Uveitis Nomenclature (SUN); posterior pole examination including vitreous cell grading measured according to SUN [3]; intraocular pressure (IOP); iris was measured using anterior segment (AS) SD-OCT Optovue RTVue (Optovue Inc, Fremont CA.), and choroidal thickness using Enhanced Depth Image (EDI)-OCT (Spectralis HRA + OCT, 870 nm; Heidelberg Engineering, Heidelberg, Germany). Visual impairment was considered mild or no visual impairment (BCVA > 20/70), moderate (BCVA < 20/70), severe (BCVA < 20/200), and blindness (BCVA < 20/400) [21]. 

Iris thickness images were taken, as described by Invernizzi [7], making a 3 mm cut from the pupillary edge to the base of the iris from the nasal and temporal sector, and without miotic drops, with dark controlled conditions with the minimal light of the equipment.

For measurement, a single horizontal line of 3 mm was drawn from the pupillary border along the pigmented posterior epithelium of the iris. The mean area was measured to give an approximate value of the average thickness of the iris. Afterward, images were exported into ImageJ medical image analysis software (ImageJ 1.45s Wayne Rasband National Institutes of Health, USA). The ImageJ software can assess the number of pixels in the selected area, setting the scale in millimeters/pixels, so the area can be calculated, expressing the result in square millimeters (mm2) or iris area (IA). Dividing this result by the previously known distance of 3 mm, a mean value of the thickness of the iris was obtained, according to the manufacturer’s manual. These measures were made separately by two specialist observers (PNL and GMHV) and masked to the SFCT of each eye.

Choroidal thickness was measured by EDI-OCT (Heidelberg Engineering, Heidelberg, Germany) and was performed by the same experienced ophthalmologist. All scans were taken without mydriasis, and in the morning, to avoid diurnal fluctuations, using the method previously described [22]. One measurement was made of choroidal thickness on subfoveal central position (SFCT), defined as the vertical distance between the hyperreflective line from the outer border of Bruch’s membrane to the hyperreflective line of the inner surface of the sclera [10]. Two different specialists (PNL and GMHV) took one measurement each [23], with at quality signal of a least of 20 dB using the OCT device´s manual measuring tool.

To control human error, an intraclass correlation coefficient (ICC) with a 95% confidence interval (CI) was calculated to measure agreement between the two observers (PNL and GMHV). The Approximate Evolution Time (AET) of FUS disease was defined as the time in FUS diagnosis in our hospital to the time of the imaging studies (anterior segment SD-OCT and EDI-OCT).

Descriptive measures and results are presented with percentages and frequencies and medians with interquartile range (IQR). For comparisons between the eye with FUS versus the HE, we used Wilcoxon´s test. Spearman´s correlation coefficient (r_s_) was analyzed between iris and choroidal thickness in FUS and HE. The same was analyzed for age, AET, and use of topical steroids. A regression analysis was performed to find the changes in SFCT (FUS and HE) by each year of age. A *p*-value < 0.05 was considered statistically significant. All analysis was made with SPSS software version 27 (IBM Corp, Armonk, NY).

## Results

Sixteen patients met the enrollment criteria. Six (37.5%) were female. The age range was 32 to 67 (median age 48 years old, IQR 41–60). Thirteen eyes (81%) had previous phacoemulsification cataract surgery with intraocular lens implantation (performed by two expert surgeons), which had a moderate to long (median 36 months, IQR 8.5–60) Approximate Evolution Time (AET); 3 patients without cataract surgery had between 3 and 7 months of AET; median time from surgery to EDI-OCT was 19 months (IQR 6.5–22.5); median time with FUS diagnosis was 30 months (IQR 5.5–57). Demographic and clinical features are described in Table 1. Topical treatment of the eyes was as follows: 8 (50%) eyes had none, 4 (25%) had 1% suspension prednisolone acetate, 3 (19%) were treated for hypertension (brimonidine + dorzolamide + timolol), and one (6%) with 1% suspension prednisolone acetate plus timolol. Visual acuity of the patients was: no visual impairment (VI) to mild in 11 (68%). The median IOP in FUS eyes was 14.5 mmHg (IQR 12–17), similar to that of the HE at 15.50 mmHg (ICR 13–17), *p* < 0.49.

**Table 1:** Demographic and clinical features of patients and their eyes presenting Fuchs uveitis syndrome (*n* = 16)

Age (median, IQR)	48 years (41–60)
Gender (M / F)	10 (63%) / 6 (37%)
Anterior Segment Signs	
Anterior Chamber Cells	9 (56%)
Koeppe and Busacca Nodules	1 (6%)
Keratic Precipitates	5 (31%)
Cataract	2 (12%)
Pseudophakia	13 (81%)
Vitreous Cells	100 (100%)
Visual Impairment LogMAR	
Mild or No VI	11 (68%)
Moderate VI	3 (19%)
Severe VI	0 (0%)
Blindness	2 (12%)
IOP mmHg, median (IQR)	14 (12–17)

F, female; IOP = intraocular pressure; IQR = interquartile range; M, male; VI = visual impairment

The ICC between the two observers in all measurements was 98.9%, with a lower 95% confidence interval of 97% in all measurements. The mean of both observations was used as the measurement.

**Fig. 1:**
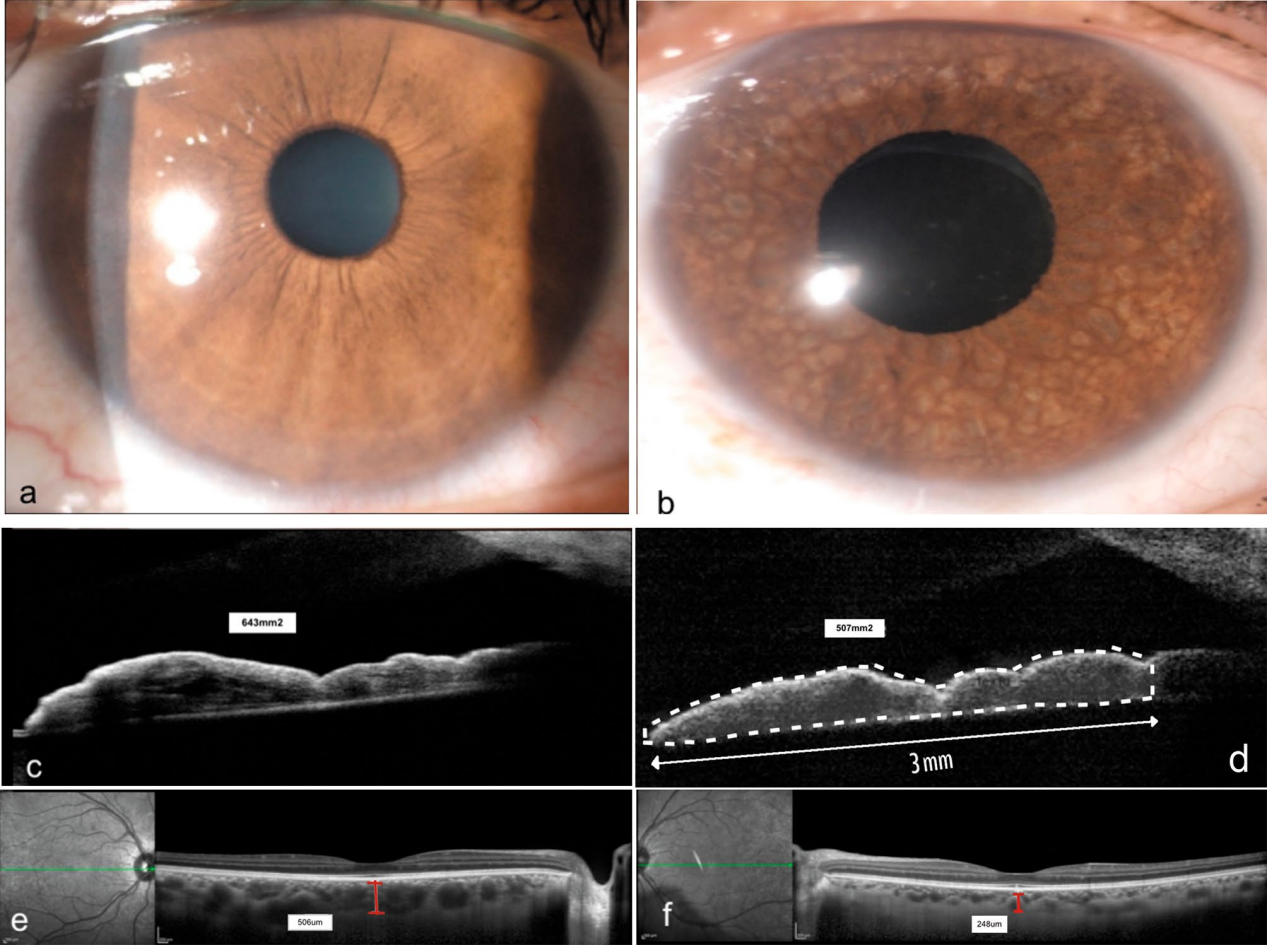
1**a**. Clinical image of the normal right eye (RE). 1**b**. Clinical image of Fuchs uveitis syndrome (FUS) left eye (LE) with pronounced iris atrophy. 1**c**. AS-OCT of RE with normal thickness iris (643mm^2^). 1**d**. Affected LE with a marked decrease in iris stroma thickness (507mm^2^), images were taken making a 3 mm cut from the pupillary edge to the base of the iris. (Technique proposed by Invernizzi [7]). Dotted white line is the iris area. 1**e**. RE Spectral domain EDI-OCT in the healthy eye; red line measures choroidal thickness on the subfoveal central position (SFCT), as the distance from the outer border of Bruch’s membrane to the inner surface of the sclera [10] with a normal choroidal thickness (506 μm). 1**f**. LE with FUS, showing a significant decrease in choroidal thickness measured with the red line (248 μm)

The total median iris area in eyes with FUS was significantly thinner than in their corresponding HE (*p* < 0.002, Table 2), and, specifically, the temporal and nasal iris areas were compromised (*p* < 0.01 and *p* < 0.001, respectively) (Fig. 1a, b, c, and d). However, when we compared the median iris area between the nasal (0.555mm^2^) and temporal (0.517mm^2^) in the FUS eyes, there was no significant difference (*p* < 0.6). The eyes with FUS had a significantly thinner SFCT than their HE counterparts (*p* < 0.0001, Fig. 1e and f).

**Table 2:** Iris and choroidal thickness measures. (*n* = 16)

	FUS eye	HE	*P*
Median iris area, mm^2^ (IQR)	*T*: 0.517 (0.464–0.635)	*T*: 0.638 (0.588–0.687)	< 0.01
	*N*: 0.555 (0.456–0.689) *p* = 0.6	*N*: 0.725 (0.648–0.769) *p* = 0.001	< 0.001
Median iris total area, mm^2^ (IQR)	*N + T 0.535* (0.466–0.629)	*N + T: 0.681* (0.627–0.738)	0.002
Median SFCT, µm (IQR)	212.25 (IQR 183-266.75)	316 (IQR 231.62–427.25)	< 0.0001

FUS = Fuchs’ uveitis syndrome; HE = healthy eye; IQR = interquartile range; *N* = nasal; *N + T* = nasal plus temporal; SFCT = subfoveal choroidal thickness; *T* = temporal;

A low correlation was found between the decreased iris and choroid thickness in FUS eyes with no statistical significance (r_s_ = 0.21; *p* = 0.4) and the same in HE (r_s_ = 0.10; *p* = 0.6). A moderate negative correlation was found between age and choroid thickness in FUS eyes (r_s_ = − 0.581; *p* = 0.018) and in HE (r_s_ = − 0.724; *p* = 0.002). (Table 3). We observed no correlation between the use of ophthalmic topical steroids and iris or choroid thickness in FUS eyes (r_s_ =0.21 *p* = 0.4; r_s_ =0.10 *p* = 0.7, respectively).

**Table 3:** Correlations values by Age, Approximate Evolution Time (AET) and Anterior Chamber Inflammation with Iris and Choroid Thickness

	Age	AET	Ach inflammation	Topic Steroid
FUS eye, Rho (p)				
Iris thickness	-0.063 (*p* = 0.8)	0.139 (*p* = 0.6)	-0.209 (*p* = 0.4)	0.21 (*p* = 0.4)
Choroid thickness	-0.581 (*p* = 0.01)	0.323 (*p* = 0.2)	0.106 (*p* = 0.6)	0.10 (*p* = 0.7)
HE, Rho (p)				
Iris thickness	-0.103 (*p* = 0.7)	0.482 (*p* = 0.058)	-	-
Choroid thickness	-0.724 (*p* = 0.002)	-0.423 (*p* = 0.1)	-	-

Rho = Spearman correlations values; *p* < 0.05

ACh = anterior chamber; AET = FUS diagnosis time in our hospital to the time of the imaging studies (anterior segment SD-OCT and EDI-OCT); FUS = Fuchs Uveitic Syndrome; HE = Healthy eye

A statistically significant difference was found between choroidal thickness in FUS/HE by age sub-groups (Table 4).

**Table 4:** Choroidal Thickness in FUS eyes and HE, grouped by age in 10-year intervals

Age groups (years)	n (%)	FUS eyes Choroidal thickness Mean ± SD (µm)	*p*	HE Choroidal thickness Mean ± SD (µm)	*p*
A (32–42)	5 (31%)	310.20 ± 100.81	With group C *p* = 0.038	407.90 ± 76.68	-
B (43–52)	5 (31%)	211 ± 33.51	-	387.10 ± 108.82	-
C (53–67)	6 (38%)	174.75 ± 81.40	-	215.16 ± 64.03	With group A *p* = 0.007; With group B *p* = 0.015

ANOVA test: choroidal thickness in FUS eyes (*p = 0.036*) and HE (*p = 0.004*); iris thickness in FUS eyes (*p = 0.4*) and HE (*p = 0.5*)

Our study derived the following regression analysis formula for choroidal thickness: FUS eyes 488.28–5.27 x Age (in years); therefore, every year, choroidal thickness decreases by 5.27 μm. In the contralateral HE, our study derived the following regression analysis formula for choroidal thickness: contralateral HE = 742.47–8.39 x Age (in years); hence, every year, choroidal thickness decreases by 8.39 μm.

When analyzing the total SFCT and the total iris area between FUS eyes with (13 patients) or without (3 patients) phacoemulsification cataract surgery, we did not find significant differences (U Mann-Whitney Test *p* < 0.9/0.8, respectively).

## Discussion

Our study found simultaneous iris and SFCT thinning in the FUS eye in most of our patients with FUS. This finding further supports the hypothesis that FUS and the associated iris and choroid thinning might have a common cause. In previous studies, a viral etiology was described [24]. However, in 2021, the SUN Working Group excluded aqueous PCR samples positive for cytomegalovirus, herpes simplex virus, and varicella zoster virus from the diagnostic criteria for FUS [3]. 

Prior studies found no gender bias in FUS [4, 5], iris [7], or SFCT [25] thinning. Therefore, the gender ratio in our study (10 males, 6 females) is unlikely to affect our results. The median age of 48 years in our sample was above other reports [6, 7, 9, 11, 25, 26] but these studies did not show an age-related progression. A possible explanation for the finding of older age in our patients could be that we do not know the precise onset of signs and symptoms of FUS; for this reason, we calculated the AET (Approximate Evolution Time) of FUS disease, defined as the time from FUS diagnosis in our hospital up to the time of the imaging studies (anterior segment SD-OCT and EDI-OCT). However, the SFCT thinning in our FUS patients started at an early age, contrary to what has been reported by other studies [11]. For this reason, we suggest that longitudinal studies be carried out to confirm the above.

We found fewer patients with inflammation in the anterior chamber with keratic precipitates, contrary to that reported by Kardes et al [8] and Özdamar et al. [26], and a higher pseudophakia rate compared with Kardes et al. [8] (81 vs. 12%, Table I). The reason is that our patients come for ophthalmologic consultation because of a decline in visual acuity caused by a cataract requiring surgery.

In our patients, the iris thickness in FUS eyes (nasal vs. temporal and total area = nasal + temporal) was significantly thinner than in the HE (Table 2). However, iris thickness measured in this study in both FUS and HE was greater compared to all previous reports [6–8]. This might be associated with our ethnic group [16]. In our experience, with darker eyes, like other Latin populations, the anterior stroma iris atrophy is more severe, so the pigmented layer turns into the dominant layer [3]. 

As in previous reports [7], we did not find any difference between the temporal/nasal iris area (*p* < 0.6) in the FUS eye. Other authors have mentioned that the reason for this is likely due to ischemia and secondary atrophy associated with FUS eyes and minor vascular irrigation in the temporal side of the iris [7, 26, 27]. In contrast, in healthy eyes, we found a greater difference in the thickness between the temporal and nasal iris area (*p* < 0.001); this could be associated with the presence of the two branches of the anterior ciliary artery in this region [7]. 

Similarly, we found that the median SFCT in FUS eyes was significantly thinner. The reason behind this may be chronic inflammation that affects choroidal perfusion and decreased vascular area volume, causing thinning of the choroid [9, 10, 25, 28]. Our SFCT measurements in FUS eyes were the thinnest of all those reported (median 212.25 μm, IQR 183-266.75 μm).

Additionally, this study is unique in that it compares iris and choroidal thickness in FUS at the same time and in the same patient, intending to identify any relationship between these two structures. It has been reported that in healthy subjects, there is a statistically significant correlation between the two [29]. 

The measurement of SFCT can have many confounding factors. Firstly, ethnic group; in the Hispanic population, values are thinner compared with other populations [17]. Secondly, age; the finding of a statistically significant moderate negative correlation between age and choroidal thickness, both in FUS and HE in our patients, is consistent with the findings of other authors in normal eyes [23, 30], who reported that, with increasing age, the choroid decreases by 3.14 μm for each year of age or about 15.6 μm for each decade of life. The age range of our patients, 32 to 67 years (median age 48 years old, 41 to 60 years), showed that SCFT decreased approximately 5.27 μm in FUS eyes and about 8.39 μm in the contralateral HE for every year of age. These results must be interpreted with caution because our study only took one measurement of the SFCT, like Ozdogan et al [23] and Lee et al. [31] and we did not measure the refractive error or the axial length. In addition, when we compared the SFCT in FUS/HE by age sub-groups, there was a significant difference between age sub-groups (in FUS eyes in group A with C; and in HE in group A with C and B with C); hence, age will probably influence choroidal thickness in the future in patients with FUS, as described in the healthy population [23]. However, there was no statistically significant difference in the iris thickness analysis in the FUS eye by age sub-groups.

Thirdly, we did not find a significant correlation between the anterior chamber cells and SFCT / iris thickness, unlike previous reports [32]. Therefore, we suggest close follow-up with these patients to detect any correlation between different stages of inflammation on the anterior chamber and the choroidal thickness. This has been described in other autoimmune uveitis, mainly Behcet [8], and Vogt-Koyanagi-Harada disease [33], in which it has been proposed that choroidal thickening could be due to increased blood flow secondary to active inflammation; this is contrasted with a thinner choroid in the presence of chronic and long-lasting inflammation that modifies choroidal perfusion, leading to atrophy of choroidal tissue and fibrosis. In addition, FUS is a peculiar form of uveitis, since inflammation is asymptomatic for the patient, the presence and severity of the inflammation vary in each appointment, and a small benefit is obtained from topical corticosteroid therapy to treat anterior chamber inflammation, making it a challenge to study prospectively [34]. 

Fourthly, cataract surgery did not alter or complicate the signs of FUS in our patients, in agreement with other authors; [35, 36] nor did it have a significant association with SCFT; it could be associated only with the inflammatory process [25]. Our patients had a median of 19 months from surgery to imaging studies, which, as seen by Zeng et al., does not influence SCFT [37]. Finally, concerning ocular hypertension, although we did not have any patients with glaucoma, it has been reported that open-angle glaucoma has no significant difference in SFCT when compared to control [38]. 

Limitations of our study include a small sample size and that we conducted a cross-sectional study, resulting in a low correlation between iris and choroid thinning in FUS eyes. However, the use of paired samples of healthy versus FUS eyes in each patient increases the power to control for interindividual variance, which, as mentioned, can vary widely. Another limitation was that we did not use miotic eye drops and we only had controlled light conditions in all patients at the moment iris thickness measurements were taken. If the results shown here can be replicated in more diverse, larger samples and a longitudinal study, a strong case can be made for searching for an inflammatory response triggered by a variety of causes. One cannot rule out the influence of embryonic development (explained because the iris and retinal pigment epithelium share the same embryonic origin) [39] and the influence of irrigation, coming together to form a synergistic syndrome.

## Conclusions

This is the first study that simultaneously evaluates iris and choroid thickness in patients with FUS and found reduced iris area and subfoveal choroidal thickness in eyes with FUS compared to the normal fellow eye. With these changes, we propose that the underlying pathophysiological causes of iris and SFCT thinning in FUS are coexisting, which may have implications for biomarkers of FUS, as well as questions about FUS pathogenesis.

## Data Availability

The datasets used and/or analyzed during the current study available from the corresponding author on reasonable request.

## Abbreviations

AET: Approximate Evolution Time
AS-OCT: Anterior Segment Optic Coherence Tomography
EDI-OCT: Enhanced Depht Image Optic Coherence Tomography
FUS: Fuchs Uveitis Syndrome
HE: Healthy eye
IA: Iris area
ICC: Intraclass correlation coefficient
IQR: Inter Quartile Range
LE: Left eye
RE: Right eye
r_s_: Spearman correlation coefficient
SFCT: subfoveal choroidal thickness
